# Supplementary material for: Implementing intravenous iron for maternal anemia in Nigeria: A qualitative study of healthcare provider experiences using the normalization process theory
Source: PLoS One. 2026 Feb 23;21(2):e0337162. doi: 10.1371/journal.pone.0337162 (PMC12928493; doi:10.1371/journal.pone.0337162)
Supplement: S1 File — (PDF) [file pone.0337162.s001.pdf]

COREQ Checklist

Consolidated criteria for reporting qualitative research

Manuscript: Implementing intravenous iron for maternal anemia in Nigeria: A qualitative study of healthcare provider experiences using the Normalization Process Theory

Item #	Topic	Guide Questions/Description	Reported on Page #
**DOMAIN 1: RESEARCH TEAM AND REFLEXIVITY**			
	Personal Characteristics		
1	Interviewer/facilitator	Which author/s conducted the interview or focus group?	Page 8
2	Credentials	What were the researcher's credentials?	Pages 1, 20
3	Occupation	What was their occupation at the time of the study?	Page 1
4	Gender	Was the researcher male or female?	Not reported
5	Experience and training	What experience or training did the researcher have?	Page 8
	Relationship with participants		
6	Relationship established	Was a relationship established prior to study commencement?	Page 7-10
7	Participant knowledge of the interviewer	What did the participants know about the researcher?	Page 10
8	Interviewer characteristics	What characteristics were reported about the interviewer?	Page 8
**DOMAIN 2: STUDY DESIGN**			
	Theoretical framework		
9	Methodological orientation	What methodological orientation was stated to underpin the study?	Pages 6, 9
	Participant selection		

10	Sampling	How were participants selected?	Pages 7-8
11	Method of approach	How were participants approached?	Pages 7-8
12	Sample size	How many participants were in the study?	Page 8
13	Non-participation	How many people refused to participate or dropped out?	Page 8
	Setting		
14	Setting of data collection	Where was the data collected?	Page 7
15	Presence of non-participants	Was anyone else present besides participants and researchers?	Not reported
16	Description of sample	What are the important characteristics of the sample?	Page 9, Table 1
	Data collection		
17	Interview guide	Were questions, prompts, guides provided? Was it pilot tested?	Page 8
18	Repeat interviews	Were repeat interviews carried out?	Not reported
19	Audio/visual recording	Did the research use audio or visual recording?	Page 8
20	Field notes	Were field notes made during/after the interview?	Page 8
21	Duration	What was the duration of the interviews?	Page 8
22	Data saturation	Was data saturation discussed?	Page 7
23	Transcripts returned	Were transcripts returned to participants for comment?	Not reported
**DOMAIN 3: ANALYSIS AND FINDINGS**			
	Data analysis		
24	Number of data coders	How many data coders coded the data?	Page 10
25	Description of the coding tree	Did authors provide a description of the coding tree?	Pages 9-10
26	Derivation of themes	Were themes identified in advance or derived from the data?	Pages 10

27	Software	What software was used to manage the data?	Page 10
28	Participant checking	Did participants provide feedback on the findings?	Not reported
	Reporting		
29	Quotations presented	Were participant quotations presented to illustrate themes?	Pages 11-18
30	Data and findings consistent	Was there consistency between data and findings?	Pages 10-18
31	Clarity of major themes	Were major themes clearly presented?	Pages 10-18
32	Clarity of minor themes	Is there description of diverse cases or minor themes?	Pages 10-18
