# Supplementary material for: Implementing intravenous iron for maternal anemia in Nigeria: A qualitative study of healthcare provider experiences using the normalization process theory
Source: PLoS One. 2026 Feb 23;21(2):e0337162. doi: 10.1371/journal.pone.0337162 (PMC12928493; doi:10.1371/journal.pone.0337162)
Supplement: S2 File — (PDF) [file pone.0337162.s002.pdf]

Qualitative questions for Normalization process theory

This qualitative study aims to:

1. Understand the implementation process by which IV was routinely operationalized by the healthcare workers at the health facilities.
2. To understand the barriers and facilitators to the implementation of IV iron in the routine healthcare system.

**Construct**	**Main Questions and Probe**
**Coherence (sense-making work):** How do participants understand and make sense of the new work methods?	1. How would you describe the purpose of using IV iron for treating maternal anemia?<br>2. In what ways is using IV iron different from previous methods of treating maternal anemia?<br>3. What do you see as the potential benefits of using IV iron in your practice?<br>4. How easy or difficult was it to understand the process/guidelines for administering IV iron?
**Cognitive Participation (relational work):** How do participants commit to and engage in the new work methods?	5. How were you involved in implementing IV iron use in your facility?<br>6. What factors motivated you to engage in using IV iron for treating maternal anemia?<br>7. Who were the key individuals who drove the adoption of IV iron in your facility? How did they influence the process?<br>8. How do you feel your colleagues have responded to the introduction of IV iron?
**Collective Action (operational work):** How are participants organised to facilitate the enacting of the new work methods**?**	9. How has the introduction of IV iron affected your daily work routines?<br>10. What new skills, knowledge or attitudes did you need to acquire to use IV iron effectively?

	11. How well does the use of IV iron fit with existing work processes in your facility?<br>12. What resources or support were most crucial in helping you implement IV iron use?<br>13. How has the use of IV iron affected your interactions with patients?
**Reflexive Monitoring (appraisal work):** How do participants appraise and reflect on the new work methods?	14. How do you evaluate the effectiveness of IV iron in treating maternal anemia?<br>15. What feedback have you received from patients about their experiences with IV iron?<br>16. Have you made any adjustments to how you use IV iron based on your experiences? If so, what were they?<br>17. How do you think the use of IV iron has impacted overall care for pregnant and postpartum women with anemia?<br>18. What challenges, if any, have you encountered in using IV iron, and how have you addressed them? (Probe about challenges from the provider and patient end)

General questions:

19. Do you feel that using IV iron has become a routine part of care for maternal anemia in your facility? Why or why not? (Probe about IV iron use beyond the lifespan of the project)
20. What do you think could be potential facilitators for implementation at other facilities if IV iron was scaled up for use in Nigeria?
21. What do you think could be potential barriers to implementation at other facilities if IV iron was scaled up for use in Nigeria?
22. What do you think can be put in place to make it work better?

23. Is there anything else you'd like to share about your experience with implementing and using IV iron for maternal anemia?
